# Lung cancer clustering by identification of similarities and discrepancies of DNA copy numbers using maximal information coefficient

**DOI:** 10.1371/journal.pone.0301131

**Published:** 2024-05-13

**Authors:** Nezamoddin N. Kachouie, Wejdan Deebani, Meshal Shutaywi, David C. Christiani

**Affiliations:** 1 Department of Mathematics and Systems Engineering, Florida Institute of Technology, Melbourne, FL, United States of America; 2 Mathematics Department, College of Science and Arts, King Abdulaziz University, Jeddah, Saudi Arabia; 3 Department of Environmental Health, Harvard School of Public Health, Boston, MA, United States of America; 4 Department of Epidemiology, Harvard School of Public Health, Boston, MA, United States of America; National Institute of Cancer Research, TAIWAN

## Abstract

Lung cancer is the second most diagnosed cancer and the first cause of cancer related death for men and women in the United States. Early detection is essential as patient survival is not optimal and recurrence rate is high. Copy number (CN) changes in cancer populations have been broadly investigated to identify CN gains and deletions associated with the cancer. In this research, the similarities between cancer and paired peripheral blood samples are identified using maximal information coefficient (MIC) and the spatial locations with substantially high MIC scores in each chromosome are used for clustering analysis. The results showed that a sizable reduction of feature set can be obtained using only a subset of locations with high MIC values. The clustering performance was evaluated using both true rate and normalized mutual information (NMI). Clustering results using the reduced feature set outperformed the performance of clustering using entire feature set in several chromosomes that are highly associated with lung cancer with several identified oncogenes.

## 1. Introduction

Lung cancer is the leading cause of cancer death in the United States [1–4]. It is the second most diagnosed cancer in both men and women in the US [1]. Over half of patients diagnosed with lung cancer die within one year of diagnosis and the 5-year survival is around 17.8% [3, 4]. Two main sub-types of lung cancer include non-small-cell lung carcinoma (NSCLC) and small-cell lung carcinoma where NSCLC accounts for about 85% of all lung cancers [2– 4]. Depending on the NSCLC stage, different treatments including surgery, radiation, chemotherapy, or targeted therapy might be considered. With the advancement of somatic genetics and biomarkers testing, specific mutations have been identified to better target treatment for individual patients [5].

An estimated 238,340 adults including 117,550 men and 120,790 women in the US will be diagnosed with lung cancer in 2023 [2, 3]. Cigarette smoking is a major risk factor for developing NSCLC. After increasing for decades, lung cancer rates are decreasing nationally as fewer people smoke cigarettes [1]. Overall incidence rates among men and women have dropped by around 2% each year since the mid-2000s. The number of new lung cancer cases diagnosed in men each year has been dropping annually since the mid1980s, while the number of new cases has been dropping in women since the mid-2000s [2]. Incidence rates are dropping faster in men than in women.

Moreover, lung cancer makes up around 25% of cancer deaths. It is estimated that 127,070 deaths from lung cancer including 67,160 men and 59,910 women will occur in the US in 2023. However, death rates for the disease have declined by 54% since 1990 in men and 30% in women since 2002 [2]. The death rates for men and women with lung cancer dropped by 5% and 4% respectively each year from 2014 to 2018. Research indicates that these declines are due to medical advances in diagnosis and treatment, fewer people smoking, and more people quitting smoking. The average age of diagnosis is 70 and people aged 65 and older are more likely to develop the disease. In comparison with white males, black males are about 15% more likely to get lung cancer. In contrast, black females are 14% less likely to get lung cancer in comparison with white females [2, 3].

The rapid progress in machine learning technologies along with the advancements in the infrastructure for information technologies such as the graphics processing unit (GPU), and the development of public databases, have made it possible to make use of large-scale data and have motivated a great deal of interest in using machine learning and artificial intelligence (AI) technologies in cancer diagnosis and clinical oncology [6–8]. Nearly 350 AI equipped medical devices have been approved in the US by the FDA [9]. Among them imaging and diagnostic technologies lead the integration of algorithms to drive clinical decision-making in healthcare. Several AI equipped medical devices have already been used for clinical applications such as diagnostics imaging. Moreover, machine learning techniques have been developed for Precision Medicine by customization and optimization of the medical care for each individual and Precision Oncology is taking advantage of advancements in the machine learning for choosing treatment options [10–12]. Machine learning algorithms have also been used for Genomic Medicine to use genomic information of individuals as part of their clinical care [12, 13].

Some blood tests are performed to detect signs of cancer such as CBC (Complete Blood Count), Electrophoresis blood test, and Tumor marker test. Moreover, some tests are performed to detect proteins or other substances made by the cancer in blood and are performed after cancer diagnosis such as Cancer antigen tests, Circulating tumor cell tests, and Genetic tests. These blood tests have been somewhat successful in diagnosing some types of cancer like prostate cancer, colon cancer, and ovarian cancer by detecting various proteins or chemicals made by cancer cells. However, these tests do not always help with cancer diagnosis as some healthy cells also make these proteins and chemicals, and some non-cancer conditions can also cause high levels of tumor markers.

The premise of future advancements in cancer research is to use blood samples to test DNA changes to detect signs of cancer in healthy people with no symptoms. The research conducted here is in-line with an active area of research to use a blood sample for testing DNA changes for cancer diagnosis. In our previous works, we investigated common CN changes among cancer population [14]. We then studied correlations between cancer and matched blood samples [15] by computing maximal information coefficient (MIC) at each spatial location (locus) of each chromosome to quantify and identify similarities between tumor and blood samples. In contrast with the Pearson’s correlation coefficient that is relevant for quantifying the strength of linear correlations, MIC can be employed to quantify either linear or non-linear correlations. MIC assumes values between zero and one where values above 0.5 demonstrate substantial correlation, and the MIC values close to one reveal strong correlations. We showed in [15] that a few chromosomes with a large set of CN changes (loci) can potentially be used to identify early signs of NSCLC. In contrast, another group of chromosomes with several CN changes (loci) are potential candidates to develop biomarkers for separating cancer from the matched blood sample. Also, we divided patients into healthy and cancer groups using 240,000 paired CN’s collected for each individual participated in the study [16].

In this work, we extend our previous studies in [15] and [16] to separate tumor samples from non-involved tissue (blood samples) using a reduced feature-set extracted from a large set of significant identified loci. The reduced set can be potentially used for separating cancer patients from healthy individuals. Our research using paired tumor-blood samples taken from lung cancer patients is an essential step towards the future advancements in cancer research using blood samples for testing DNA changes to detect signs of cancer in people with no symptoms. We demonstrated potential differences and similarities in DNA copy numbers in tumor sample in comparison with blood sample that can be potentially used in future to develop cancer tests for lung cancer diagnosis using patient’s blood sample.

## 2. Data description

A set of 63 early stage (stage 1 and 2) non-small cell lung cancer (NSCLC) patients were prospectively enrolled in the Boston Lung Cancer Study at the Massachusetts General Hospital (MGH), Boston, MA [17, 18]. A snap-frozen tumor sample was gathered for each patient during biopsy or surgery in addition to a blood sample. Table 1 shows a snippet of the CN scores ranging from one to three. Deleted CNs have values below two while values above two indicate gained CNs.

**Table 1:** A snapshot of the DNA copy number values for chromosome 1.

Observation	True Group	Location	Location	Location	. . .	Location	Location
Number		1	2	3		19872	19873
1	cancer	2.22	1.66	1.66		1.85	1.85
2	blood	1.82	1.82	1.82	. . .	1.55	1.89
3	cancer	1.7	1.7	1.7	. . .	1.76	1.76
4	blood	1.88	1.86	1.86	. . .	2.15	2.15
5	cancer	2.2	2.2	2.2	. . .	2.25	2.3
6	blood	1.9	1.9	1.9	. . .	2.32	2.32
7	cancer	2.19	2.02	2.02	. . .	1.95	1.95
8	blood	1.86	1.9	1.9	. . .	2.06	2.06
9	cancer	1.96	2.01	2.01	. . .	2.19	2.08
10	blood	2.16	2.16	2.16	. . .	2.25	2.25
11	cancer	1.47	1.47	1.52	. . .	1.63	2.12
12	blood	2.61	2.61	2.61	. . .	1.71	1.71
.	.	.	.	.	. . .	.	.
125	cancer	2.07	2.07	2.07	. . .	1.96	1.96
126	blood	2.44	2.44	2.44	. . .	2.16	2.16

## 3. Methods

Mass General Brigham IRB committee approved this study and participants provided written informed consent. Lung cancer clustering was performed in the previous work [16] using chromosome-wide spatial copy number variations among paired cancer and non-involved (blood) samples. Kernel K-means, a nonlinear clustering method, was applied to separate cancer from normal samples in each chromosome using DNA CN’s. A snippet of CN values for chromosome one obtained using paired cancer-blood samples for 63 patients is shown in Table 2. It should be noticed that copy numbers are obtained for all spatial locations (loci) on a chromosome and in turn several thousand CN’s are collected for each chromosome. Therefore, the corresponding feature space consisting all copy numbers for each chromosome is highly dimensional. For example, there are 19,873 CN’s collected for chromosome one, which forms a 19,873-dimensional copy number space [16].

**Table 2:** Structure of the data for chromosome one (as an example).

Patient	Location 1		Location 2		. . .	Location 19,873	
	Cancer Sample	Blood Sample	Cancer Sample	Blood Sample		Cancer Sample	Blood Sample
Patient 1	2.22	1.82	1.66	1.82		1.85	1.89
Patient 2	1.7	1.88	1.7	1.86		1.76	2.15
Patient 3	2.2	1.9	2.2	1.9		2.3	2.32
Patient 4	. . . .	. . . .	. . . .	. . . .		. . . .	. . . .
Patient 5	. . . .	. . . .	. . . .	. . . .		. . . .	. . . .
.	.	.	.	.		.	.
Patient 63	2.07	2.44	2.07	2.44		2.16	2.16

It is essential to reduce the dimensions of the chromosomal feature space to improve the discriminant power. Hence, for feature reduction, the significant features must be identified and extracted from the feature space. To this end, we quantify the similarities and discrepancies between cancer and paired peripheral blood samples using a correlation measure, so called maximal information coefficient (MIC) [19]. In the previous work [15], correlations between cancer and matched blood samples were studied by quantifying MIC at each spatial location of each chromosome. In this work, an upper and a lower threshold will be set for identifying significant chromosomal features with high discriminant power of detecting cancer from healthy. The reduced feature set will then be used for grouping individuals to the two groups of cancer and healthy.

MIC takes a value between zero and one and can identify linear and nonlinear associations [19]. MIC values above 0.5 demonstrate substantial correlation (similarities) while MIC values below 0.2 indicate low or no correlation (discrepancies) between the samples. MIC values that exceed the upper threshold represent high correlation between cancer and non-involved tissue and can be considered as early indicators of NSCLC. MIC values that fall below the lower threshold represent low correlation between cancer and non-involved samples and are relevant for developing biomarkers to distinguish cancer from matched blood samples [15].

MIC for the *j*^*th*^ location for a typical chromosome is calculated using [20]:

$$MIC_{j}=\max\{\frac{I(blood_{j},cancer_{j})}{\log_{2}\min\{n_{blood_{j}},n_{cancer_{j}}\}}\},j=1,2,\cdots ,m$$ (1)

where *m* is the total number of locations and

$$\begin{array}{l} I(blood_{j},cancer_{j})=H(blood_{j})+H(cancer_{j})-H(blood_{j},cancer_{j})\\ \;=\sum_{w=1}^{nblood_{j}}p(blood_{j_{w}})\log_{2}\frac{1}{p(blood_{j_{w}})}\\ \;+\sum_{s=1}^{n_{cancer_{j}}}p(cancer_{j_{s}})\log_{2}\frac{1}{p(cancer_{j_{s}})}\\ \;-\sum_{w=1}^{n_{blood_{j}}}\sum_{n_{cancer_{j}}}^{s=1}p(blood_{j_{w}},cancer_{j_{s}})\log_{2}\frac{1}{p(blood_{j_{w}},cancer_{j_{s}})} \end{array}$$ (2)

where *p*(*blood*_*j*_, *cancer*_*j*_) is the joint probability distribution of *j*^*th*^ blood and *j*^*th*^ cancer samples respectively, *p*(*blood*_*j*_) and *p*(*cancer*_*j*_) are the marginal distributions of *j*^*th*^ blood sample and *j*^*th*^ cancer sample respectively, *nblood*_*j*_ and *ncancer*_*j*_ are the number of bins in the partitions associated with the blood and cancer respectively and *nblood*_*j*_ ×*ncancer*_*j*_<*B*(*n*) where *B* (*n*) = *n*^0.6^, and *n* is the sample size (*n* = 63). After calculating MIC values at each locus of every chromosome, we identify spatial locations with considerably high correlation scores between cancer and blood samples. We locate MIC values greater than a threshold *γ*.

Let *v* be the number of selected locations with correlation values greater than *γ* in a given chromosome, where *v < m*. Then, we use kernel K-means clustering method which is a nonlinear extension of K-means clustering and can separate both linearly and non-linearly separable clusters [21, 22]. Kernel K-means clustering projects the data into a higher-dimensional feature space using a nonlinear function. In this way, projected points will be linearly separable in the transformed space. Let {*x*1,*x*2,…,*xn*} be a set of *n* data points (*n* = 126) with *v*-dimension, *K* be the number of clusters, *π*_*k*_ be the cluster *k*, $\{\pi_{k}{\}}_{k=1}^{K}$ be a partitioning of points into *K* groups, and *φ* be a non-linear function. For each element of the kernel matrix, Mij=φ(xi)×φ(xj),i,j=1,2,…,n, where *φ*(*x*_*i*_) and *φ*(*x*_*j*_) denote the data points *x*_*i*_ and *x*_*j*_ in the transformed space respectively. The Euclidean distance from each data point to a cluster center *μ*_*k*_ is computed in the transformed space for all *K* clusters by:

$$\begin{array}{r} \phi (x_{i})-\mu_{k}=\phi (x_{i})-\sum_{x_{i}\in \pi_{k}}\frac{\phi (x_{i})}{\left|\pi_{k}\right|}=\phi (x_{i})\cdot \phi (x_{i})-\frac{2\sum_{x_{j}\in \pi_{k}}\phi (x_{i})\cdot \phi (x_{j})}{\left|\pi_{k}\right|}\\ +\frac{2\sum_{x_{j},x_{c},\in \pi_{k}}\phi (x_{j})\cdot \phi (x_{c})}{{\left|\pi_{k}\right|}^{2}} \end{array}$$ (3)

where |*π*_*k*_| is the number of elements in cluster *π*_*k*_. Each data point will be assigned to the closest cluster based on calculated distances between the point and cluster centers. A new cluster center *μ*_*k*_ is obtained for cluster *k* by averaging Euclidean distance of all elements that were assigned to cluster *π*_*k*_ in the transformed space in the previous iteration:

$$\mu_{k}=\underset{x_{i}\in \pi_{k}}{\sum }\frac{\phi (x_{i})}{\left|\pi_{k}\right|},\;k=1,2,\ldots ,K$$ (4)

This process will be repeated until |*μ*_*k*,*t*_ − *μ*_*k*,*t*−1_| *< ε* for all *K* clusters, where *μ*_*k*,*t*_ is the center of cluster *k* in the current iteration *t* and *μ*_*k*,*t*−1_ is the center of cluster *k* in the previous iteration *t* − 1.

## 4. Results

Lung cancer data contains copy numbers for pair cancer-blood (non-involved) samples for 22 chromosomes (excluding sex chromosome) for 63 patients. The correlation measure, i.e. MIC, was obtained at each location for each chromosome. To improve the discriminant power of chromosomes, MIC values above upper threshold were located. A high MIC score at an identified location indicates high similarity between blood (non-involved) and cancer samples. This results in reducing the copy number feature space dimensionality, and hence the unlabeled copy numbers in each chromosome form a *126×T* feature matrix, where *126* is the total number of samples (63 paired samples), and *T* is the number of identified spatial locations with MIC score above threshold in the given chromosome. Notice that MIC score at each locus (chromosome location) shows the similarity between CN’s in two groups of cancer and control (63 paired samples).

To cluster 126 unlabeled samples into two groups, kernel K-means is applied to the reduced feature matrix of each chromosome separately. In a perfect world, 63 cancer samples will be grouped together in one cluster and 63 non-involved samples will be clustered together in a different group. In practice however, each cluster contains a combination of cancer and non-involved samples. The clustering performance is evaluated by two methods; 1) quantifying the true rate of recognized cancer samples. 2) computing normalized mutual information (NMI). NMI provides a value between zero and one, where the higher values of NMI indicate better clustering results. High NMI value means that a substantial proportion of data points are grouped in the right clusters.

Clustering results for each chromosome are summarized in Table 3. As it can be seen in the table, 63 paired samples are once clustered using chromosome-wide CN’s (entire feature-set), and then using only selected CN’s (reduced feature-set) at the identified spatial locations with high MIC scores. The table shows the results for three different clustering set-ups using: (a) All spatial locations of a chromosome, (b) Selected features with MIC values greater than 0.65, and (c) Selected features with MIC values greater than 0.52. The number of selected features in each set-up, along with NMI and true rate are shown in the table.

**Table 3:** Kernel K-means clustering results for 22 chromosomes using (a) All spatial locations in each chromosome, (b) Selected features with MIC values greater than 0.65, and (c) Selected features with MIC values greater than 0.52.

Clustering using all features				Using features with MIC *>*0.65			Using features with MIC *>*0.52		
Chr. #	# Features	NMI	True Rate	# Features	NMI	True Rate	# Features	NMI	True Rate
1	19873	0.0151	0.5714	4	0.0002	0.5079	131	0.0030	0.5317
2	22215	0.0119	0.5635	16	0.0117	0.5635	392	0.0208	0.5794
3	18381	0.0597	0.6429	5	0.0275	0.5952	226	0.0666	0.6508
4	19067	0.0224	0.5873	11	0.0018	0.5238	233	0.0221	0.5873
5	17165	0.0311	0.6032	0	0	0	56	0.0066	0.5476
6	17147	0.0326	0.6032	2	0.0037	0.5317	67	0.0224	0.5873
7	13951	0.0118	0.5635	2	0.0066	0.5476	153	0.0047	0.5397
8	14840	0.0788	0.6587	0	0	0	21	0.0422	0.6190
9	11941	0.0599	0.6429	0	0	0	93	0.0544	0.6349
10	14279	0.0068	0.5476	0	0	0	52	0.0029	0.5317
11	13307	0.009	0.5556	3	0.0066	0.5476	189	0.0120	0.5635
12	13061	0.0048	0.5397	6	0.0016	0.5238	217	0.0264	0.5952
13	11118	0.0532	0.6349	2	0.0366	0.6111	89	0.0264	0.5952
14	8181	0.0149	0.5714	0	0	0	85	0.0018	0.5238
15	7014	0.0222	0.5794	0	0	0	47	0.0030	0.5317
16	7024	0.0151	0.5714	0	0	0	21	0.0002	0.5079
17	4854	0.0539	0.6349	3	0.005	0.5397	49	0.0031	0.5317
18	8149	0.0158	0.5714	0	0	0	5	0.0691	0.6508
19	2693	0.0017	0.5238	0	0	0	2	0.0915	0.6746
20	5838	0	0.5	0	0	0	28	0.0002	0.5079
21	3936	0.0186	0.5794	0	0	0	25	0.0090	0.5556
22	2520	0.0233	0.5873	0	0	0	13	0.0256	0.5873

### 4.1. Clustering analysis: Using all CNs of a chromosome

In this set-up, the clustering analysis is performed using the *126 × L* feature matrix, where *L* is the chromosome length (number of spatial locations in the chromosome) depicted in the second column in Table 3. For instance, to identify cancer from normal in the chromosome one, entire feature-set holding 19,873 features (chromosome-wide copy numbers) of paired cancer-blood subjects is used for clustering. Clustering performance yields a true rate of 56% and NMI value of 0.012. Considering very high dimensional feature space of each chromosome, a subset of features in each chromosome is used to discriminate cancer from blood samples as follows below.

### 4.2. Clustering analysis: Using selected CNs with MIC values above a set threshold

To reduce the dimensionality of feature set, clustering of cancer and blood samples is performed using a *126 × T* feature matrix in each chromosome, where *T* is the number of identified spatial locations in the chromosome with MIC score above a set threshold. Once, a threshold of 0.65 is set to identify CN locations with strong correlations between cancer and paired blood subjects. The kernel K-means clustering analysis is then performed using the reduced feature set with corresponding MIC values higher than 0.65. In this way, a very small number of highly selective CN locations will be identified demonstrating strong correlations between cancer and blood subjects at the given locations. For example, only 3 locations out of 4854 that have MIC score above 0.65 in chromosome 17 are used for clustering which provides 99.9% dimensionality reduction. Notice that in the previous work [14], amplified copy number regions that were associated with new oncogenes were found in chromosomes 17. Therefore, identifying locations with high MIC scores in a chromosome could potentially improve the diagnostic power of CN’s.

### 4.3. Performance-difference and adjusted performance-difference

Performance difference, *PD* is computed using:

$$PD={TR}_{EFS}-{TR}_{RFS}$$ (5)

where ***TR***_***EFS***_ is true rate (TR) obtained using entire feature-set (EFS), and ***TR***_***RFS***_ is true rate obtained using reduced feature-set (RFS). Adjusted performance difference, ***APD***, is calculated by:

$$APD=\frac{{TR}_{EFS}-{TR}_{RFS}}{{TR}_{EFS}}$$ (6)

In this way, the positive values of ***PD*** and ***APD*** represent improved performance using RFS in comparison with using EFS while their negative values represent declined performance using RFS.

### 4.4. Comparing clustering analysis performance: Reduced feature-set vs. entire feature-set

Setting the high threshold of 0.65 leads to selection of very few features. For example, there were only 4 (out of 19873), 16 (out of 22215), 5 (out of 18381), and 2 (out of 17147) locations with MIC scores above 0.65 in chromosomes 1, 2, 3, and 6, respectively. This provides a sizable reduction of feature space with comparable clustering results that was obtained using entire feature-set (Table 3). However, in several chromosomes including chromosome 5, 8, 9, 10, 14, 15, 16, 18, 19, 20, 21, and 22, no location with MIC score above 0.65 was identified. Hence, a new threshold was set to ensure that at least two locations with MIC value above the threshold are identified on each chromosome. Using an ad-hoc learning approach, the threshold of 0.52 was determined to satisfy the constraint. As it can be seen in Table 3, all 22 chromosomes have two or more identified locations with MIC score above 0.52. The number of identified locations with MIC above threshold varies from 2 (in chromosome 19) to 392 (in chromosome 2). For instance, 56, 21, 93, and 52 locations with MIC score above 0.52 were identified in chromosomes 5, 8, 9, and 10, while no location with MIC score above 0.65 was identified in these chromosomes.

**Fig 1:**
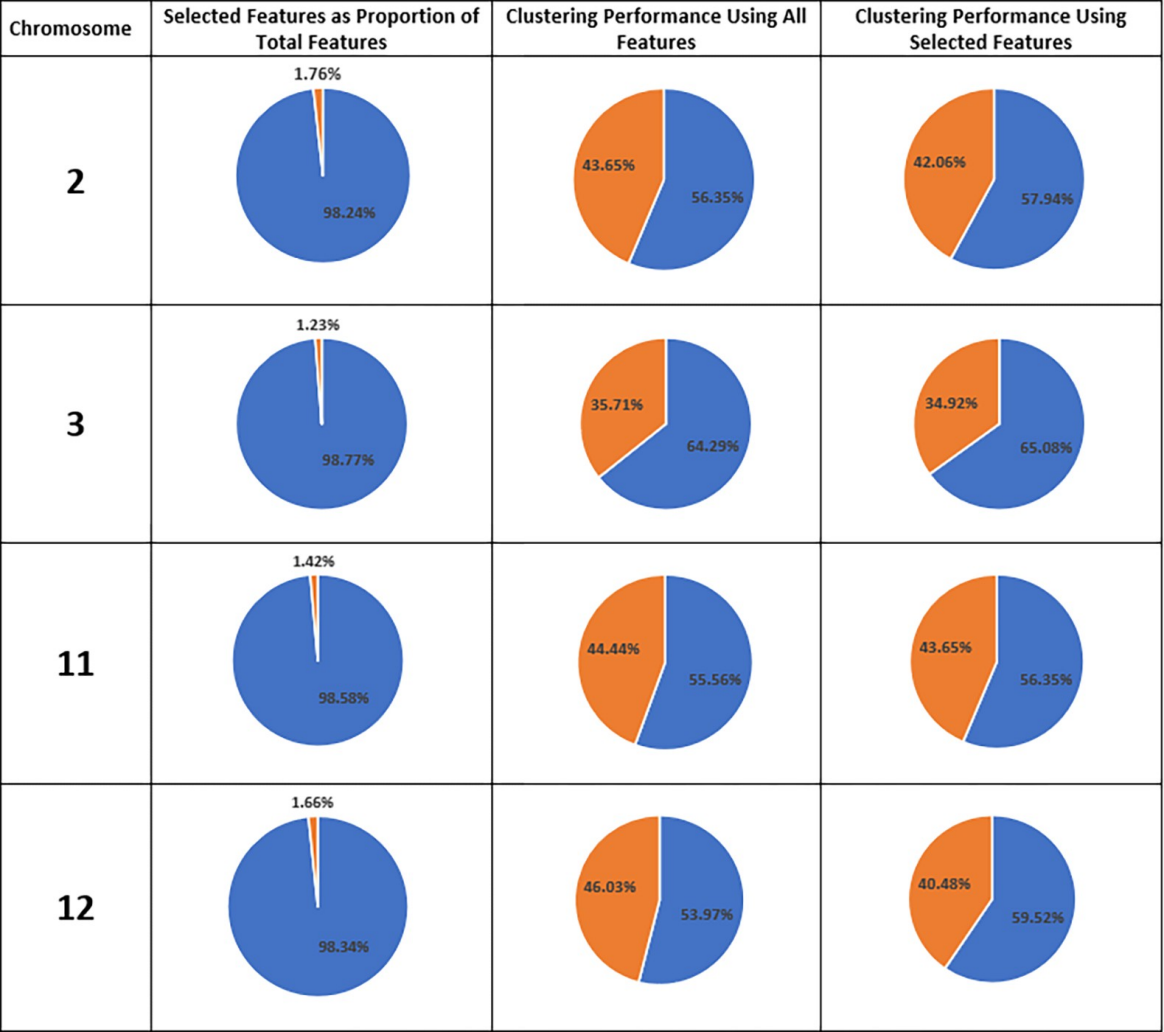
Chromosome number (first column) and proportional size of reduced feature-set in comparison with the size of the entire feature-set (second column). Clustering performance using entire feature-set (third column) and reduced feature-set (forth column). True rate (blue) and false identification (orange) based on clustering result (columns 3 and 4).

**Fig 2:**
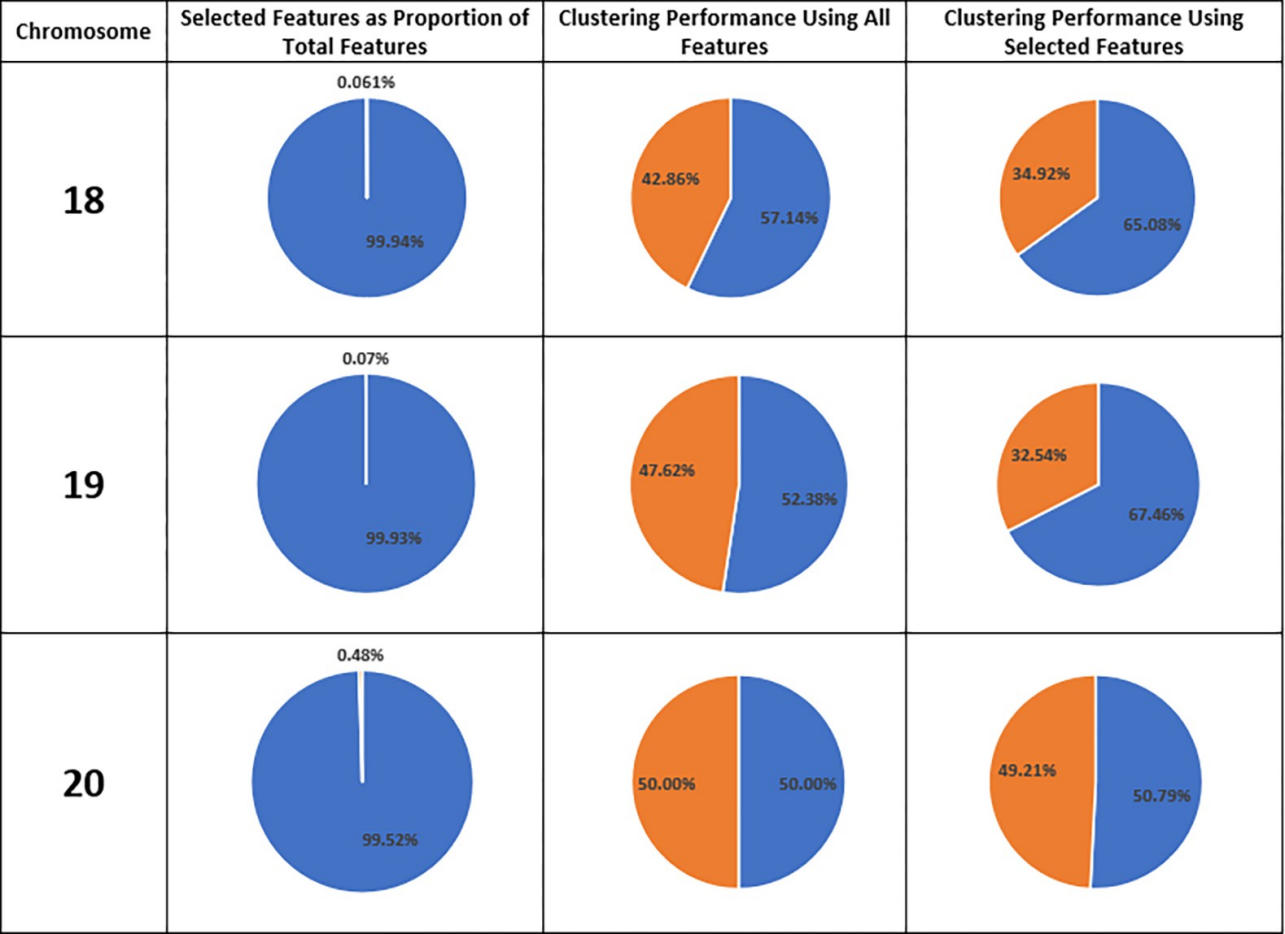
Chromosome number (first column) and proportional size of reduced feature-set in comparison with the size of the entire feature-set (second column). Clustering performance using entire feature-set (third column) and reduced feature-set (forth column). True rate (blue) and false identification (orange) based on clustering result (columns 3 and 4).

As represented in Table 3, clustering using the reduced feature-set of CN locations with MIC score above 0.52 provides comparable performance for 9 out of 22 chromosomes (about 41% of chromosomes) summarized in Table 4, and substantially better performance (10% or higher) for 3 out of 22 chromosomes (about 14% of chromosomes) summarized in Table 5. Using MIC score to identify CN locations with high correlation between cancer and control produces a reduced feature-set with considerably smaller size. Depicted in Fig 1, the proportional size of reduced feature-set ranges from 0.06% to 1.76% of that of entire feature-set. As we can see in Figs 1 and 2, and Table 5, only 2 (out of 2693) locations are identified in chromosome 19 and used for clustering, while the adjusted performance-difference and performance-difference are +29% and +15.1% respectively. Similarly, using only 5 (out of 8149) locations in chromosome 18, RFS outperforms the EFS in clustering performance yielding +14% APD and +8% PD. Overall, for six chromosomes (Table 6) clustering performance has improved with APD from +1% to +29%. Clustering performance using RFS has declined only for 10 chromosomes with PD from -2% to -10% (APD from -4% to -16%). However, yet the RFS has a significant size reduction (Figs 1 and 2, and Table 3) that can justify using selected CN locations for clustering.

**Table 4:** Kernel K-means clustering with comparable performance using reduced feature-set vs. entire feature-set.

Chr. #	Using all features		Using features with MIC *>*0.52	
	No of features	True Rate	No of features	True Rate
2	22215	0.564	392	0.579
3	18381	0.643	226	0.651
4	19067	0.587	233	0.587
6	17147	0.6032	67	0.5873
9	11941	0.643	93	0.635
10	14279	0.548	52	0.532
11	13307	0.556	189	0.563
20	5838	0.500	28	0.508
22	2520	0.587	13	0.587

**Table 5:** Kernel K-means clustering with considerable improved performance using reduced feature-set vs. entire feature-set.

Chr. #	Using all features		Using features with MIC values *>*0.52		Improvement %
	No of features	True Rate	No of features	True Rate	
19	2693	0.524	2	0.675	29
18	8149	0.571	5	0.651	14
12	13061	0.540	217	0.595	10

**Table 6:** Kernel K-means clustering with improved performance using reduced feature-set vs. entire feature-set.

Chr. #	Using all features		Using features with MIC values *>*0.52		Improvement %
	No of features	True Rate	No of features	True Rate	
19	2693	0.524	2	0.675	29
18	8149	0.571	5	0.651	14
12	13061	0.540	217	0.595	10
2	22215	0.564	392	0.579	3
20	5838	0.500	28	0.508	2
3	18381	0.643	226	0.651	1
11	13307	0.556	189	0.563	1

**Fig 3:**
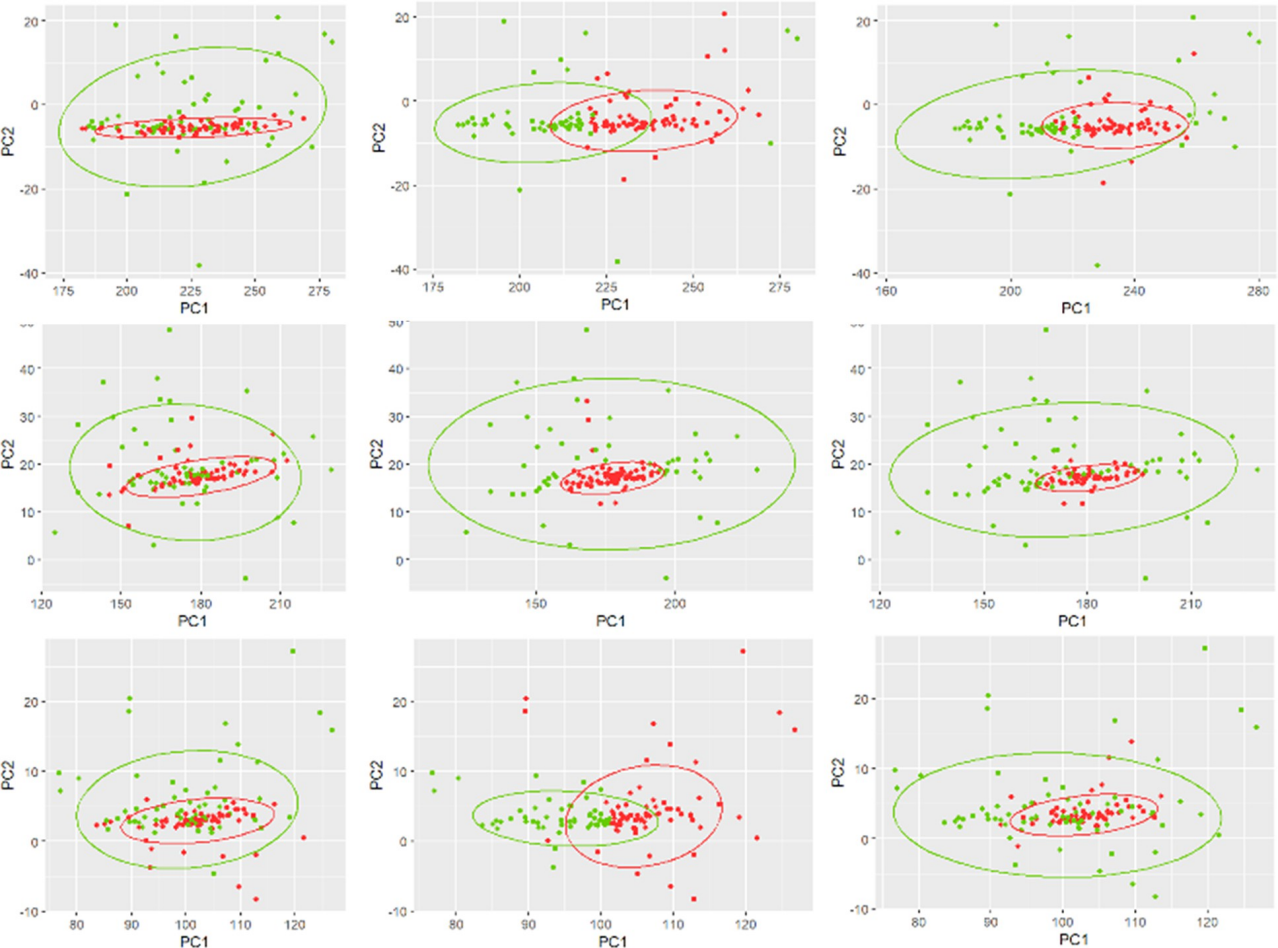
**Control (green) vs. cancer (red).** True groups (column 1), clustering results using entire feature-set (column 2), and clustering results using reduced feature-set (column 3) for chromosome 12 (row 1), chromosome 18 (row 2), and chromosome 19 (row 3).

Fig 3 displays the original groups with true labels, clustering results using EFS, and clustering results using RFS for chromosome 12, 18, and 19. The noticeable overlapping of cancer and control clusters using true labels (column 1) is clearly visible. In all three chromosomes, cancer cluster is contained inside the control group that makes the discrimination between cancer and control groups very challenging. Although, clustering using EFS could not separate the clusters, clustering using RFS could separate a subset of control group as cancer group in these chromosomes. Notice that these are the same chromosomes with the highest improvement of clustering performance using RFS with APD of +29% in chromosome 19, +14% in chromosome 18, and +10% in chromosome 12.

**Fig 4:**
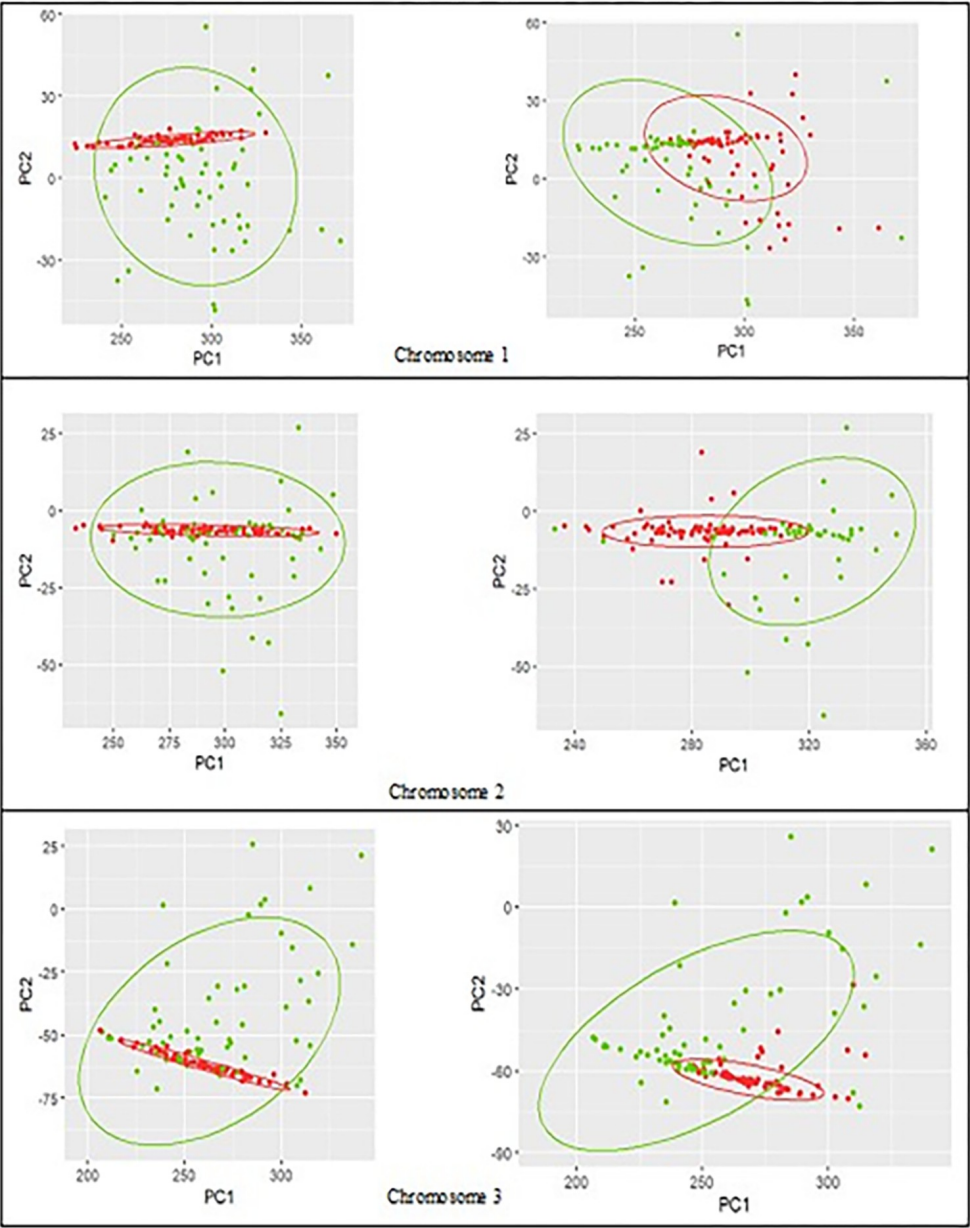
**Control (green) vs. cancer (red) groups for chromosomes 1 to 3.** True labels (left column); the assumed labels obtained by clustering using the reduced feature-set (right column).

**Fig 5:**
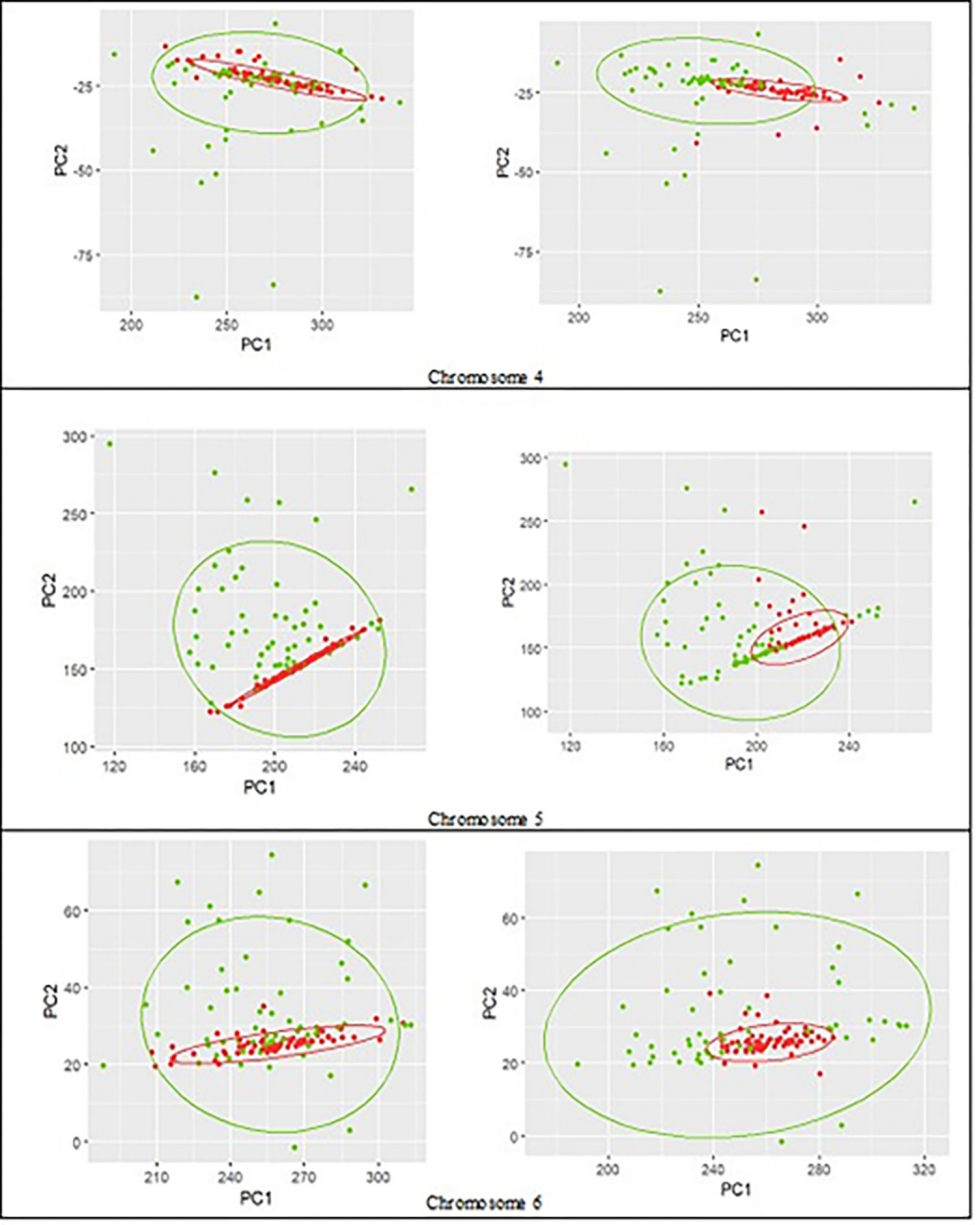
**Control (green) vs. cancer (red) groups for chromosomes 4 to 6.** True labels (left column); the assumed labels obtained by clustering using the reduced feature-set (right column).

**Fig 6:**
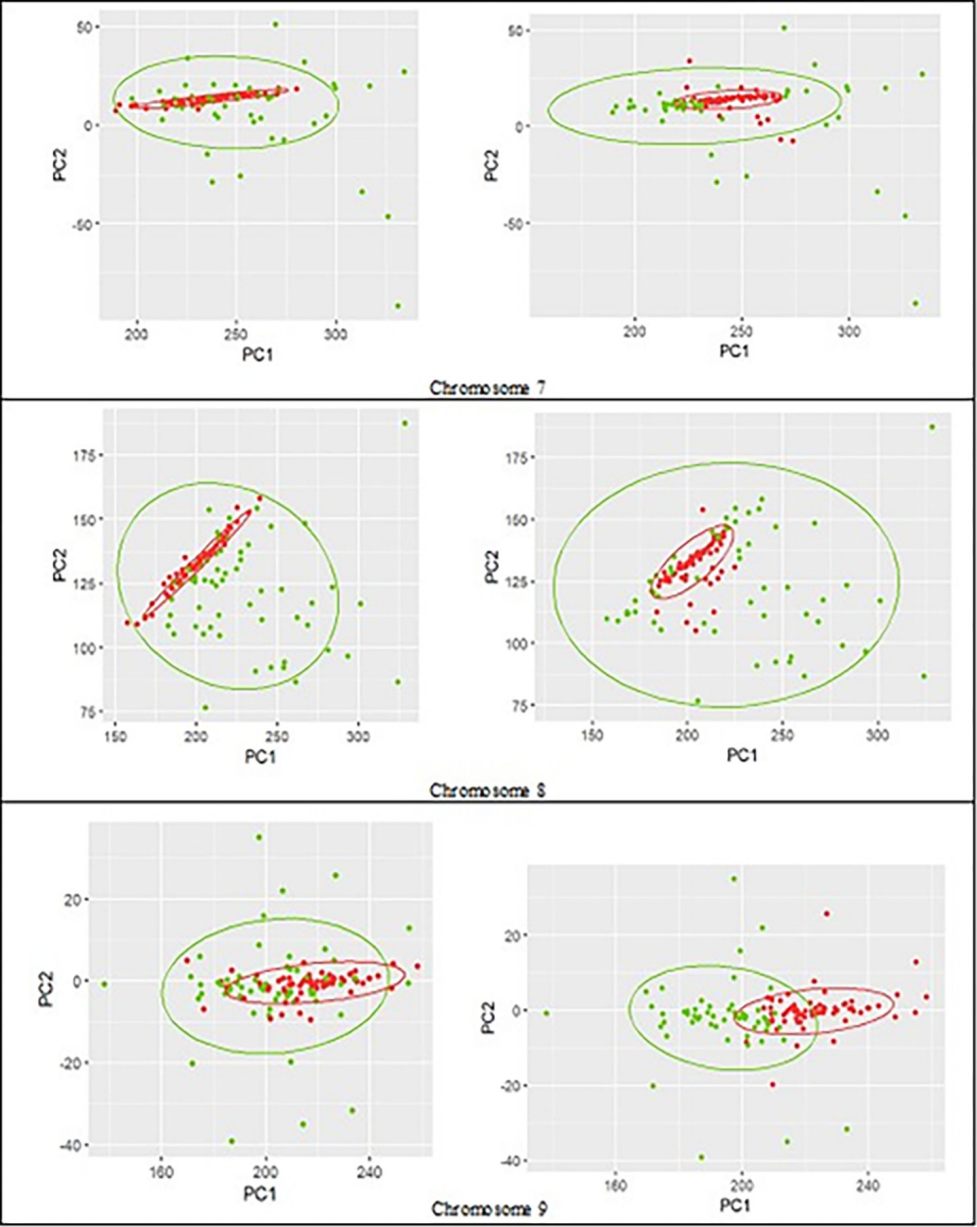
**Control (green) vs. cancer (red) groups for chromosomes 7 to 9.** True labels (left column); the assumed labels obtained by clustering using the reduced feature-set (right column).

Figs 4–6 show the true clustering labels for cancer and blood groups (left) vs. clustering results using reduced feature-set (right) for chromosomes 1 to 9 with the true rate ranges from 53% in chromosome 1 to 65% in chromosome 3, and the APD ranges from -9% in chromosome 5 to +3% in chromosome 2. The clustering performance with the TR of about 50% (NMI about zero) is due to fact that the cancer group is contained inside the control group, and it makes it very challenging to separate the two groups. Nevertheless, among the chromosomes depicted in Figs 4–6, the overall performance using RFS has only declined by up to a maximum of 5.6%, while for several chromosomes the clustering performance using RFS is comparable with the results achieved using EFS, and for chromosomes 2 and 3, the performance has improved.

Further, we compared the proposed Kernel K-means with K-means and Fuzzy c-means. K-means is a the most widely used clustering algorithm. It is an efficient centroid-based algorithm that is suitable for clustering large datasets, but it is sensitive to outliers. In contrast with K-means which is a hard-clustering technique, Fuzzy c-means is a soft-clustering method. In soft clustering, a data point may belong to different cluster with different likelihoods, rather than being assigned a hard cluster label. For most chromosomes, the proposed method outperformed both K-means and Fuzzy c-means techniques. Listed in Table 7, for five chromosomes, K-means performed better, and for three chromosomes, Fuzzy c-means outperformed the other methods. The performance of all three clustering methods for remaining 14 chromosomes are shown in Table 8. For the chromosomes listed in this table, the Kernel K-means using entire feature-set outperformed one or both K-means and Fuzzy c-means. We should point out that, for two chromosomes in Table 7, i.e., chromosomes 18 and 19, the proposed Kernel K-means method using the reduced feature-set outperformed the other methods with True rates of 0.651 and 0.675 respectively.

**Table 7:** Chromosomes that K-means or Fuzzy c-means performed better.

	Using all features		
	True Rate		
Chr. #	Kernel	K-means	Fuzzy
	K-means		c-means
1	0.5714	0.6587	0.6349
5	0.6032	0.6111	0.7063
7	0.5635	0.6111	0.6190
8	0.6587	0.6587	0.6667
9	0.6429	0.6508	0.6429
18	0.5714	0.6111	0.5952
19	0.5238	0.5556	0.5317
20	0.5000	0.5952	0.5794

**Table 8:** Clustering performance of Kernel K-means in comparison with K-means and Fuzzy c-means.

	Using all Features		
	True Rate		
Chr. #	Kernel	K-means	Fuzzy
	K-means		c-means
2	0.5635	0.5317	0.5317
3	0.6429	0.6270	0.5476
4	0.5873	0.5714	0.5714
6	0.6032	0.5238	0.5238
10	0.5476	0.5317	0.5000
11	0.5556	0.5317	0.5317
12	0.5397	0.5079	0.5238
13	0.6349	0.6270	0.6270
14	0.5714	0.5714	0.5317
15	0.5794	0.5794	0.5397
16	0.5714	0.5238	0.5238
17	0.6349	0.5556	0.5238
21	0.5794	0.5714	0.5635
22	0.5873	0.5556	0.5159

## 5. Discussion

Overall, thresholding MIC to obtain a reduced feature-set provides a sizable reduction in number of CN locations to distinguish cancer from control. The number of CN locations ranges from a minimum of 2,520 in chromosome 22 to a maximum of 22,215 in chromosome 2. The proportion of reduced set depends on the number of CN locations with MIC above 0.52 that was learned using an ad-hoc approach to identify a minimum of two CN locations in a given chromosome. The size of RFS is only a small fraction of the EFS and varies from a minimum of 0.06% for chromosome 18 to a maximum of 1.76% in chromosome 2. It means only 5 CN locations from 8,149 in chromosome 18 and 392 locations from 22,215 in chromosome 2 are identified and used for clustering. Among all, clustering results of chromosomes 19, 3, and 18 achieved the highest true rates of 67%, 65%, and 65%. Our results are noticeable as it has been shown in previous works that 3q is among the most commonly cited amplifications and 3p and 19p are among most common deletions [23–28]. Partial deletion of 3p has been reported in almost all analyzed NSCLCs [29, 30], and contains numerous genes including FHIT (3p14.2), RASSF1 (3p21.3), TUSC2 (FUS1, 3p21.3), SEMA3B (3p21.3), SEMA3F (3p21.3) and MLH1 (3p22.3) where allelic imbalance of FHIT is associated with chromosomal deletions [31, 32], RASSF1 and MLH1 are inactivated by promoter hypermethylation [33–35], TUSC2 [36], SEMA3F and SEMA3B transcripts [37] are recurrently underrepresented in lung cancers and the SEMA3s were found to be targets of TP53 [38] could be potentially activated during DNA damage. Moreover, 3p, 18p, and 19p are among the most frequent loss of heterozygosity that has been reported to occur on chromosome arms [26, 39, 40].

The clustering performance using RFS has improved in chromosome 12 yielding 60% true rate with APD of +10% indicating a net gain in PD of +6%. In lung cancer, KRAS at 12p12.1 is frequently mutated. Further, among the most important factors for lung tumor growth and proliferation the ERBB family coded by the genes including ERBB3 in 12q13 [30, 41].

To further improve the clustering performance, our future research will be focused to implement an ensemble clustering method using the proposed method and Fuzzy c-means, since it performed better on clustering of a few chromosomes as a soft clustering technique.

## 6. Conclusion

The survival rate of lung cancer, as the second most diagnosed cancer and the first cause of cancer related death in the US, is low. Early detection is critical as patient survival rate is low and recurrence rate is high. Copy number (CN) changes have been broadly investigated to identify CN amplifications and deletions associated with the cancer and can be potentially used for cancer diagnosis in future. Lung cancer data used in this project contains CN pairs for cancer and blood (non-involved) samples at each location for each chromosome for 63 subjects. In this research, the similarities between cancer and paired peripheral blood samples are identified using maximal information coefficient (MIC) and the spatial locations with substantially high MIC scores in each chromosome are used for clustering analysis. Identifying the locations with high similarities between cancer and healthy tissues in each chromosome, can potentially help with early diagnosis, treatment, and prevention of cancer. The outcomes of this research can be summarized as:

- Identifying CN locations with high correlations between cancer and control with MIC above a set threshold.
- Substantial feature reduction using a subset of CN locations with high MIC score.
- Improved clustering performance in several chromosomes that are associated with oncogenes.

Separating cancer from control is a very challenging clustering task because the cancer group coincides in the control group. In several chromosomes, reducing the copy number feature space dimensionality led to obtain comparable clustering results in comparison with the results obtained using the entire feature set. Moreover, using a small set of CN locations with high MIC exhibited improved discrimination power in some chromosomes. The highest true rate was achieved in chromosomes 19 and 18, where reduced feature set contained only 2 and 5 CN locations in these chromosomes respectively. The results suggest the identification of a handful of CN locations in each chromosome may improve the discrimination power of cancer from healthy tissue. Therefore, our future research will be focused on identifying a customized MIC threshold for each chromosome to adaptively limit the number of identified locations with high MIC scores in each chromosome.

Some blood samples are tested for cancer diagnosis by looking for signs of cancer including [42–44]:

1. Complete blood count (CBC): It measures the amount of each type of blood cell in a blood sample to diagnose blood cancer.
2. Test for blood proteins: An electrophoresis blood test looks at the various proteins in the blood sample to find those made by the immune system. This test is helpful in diagnosing multiple myeloma.
3. Tumor marker: Use a blood sample to look for chemicals made by cancer cells. These tests do not always help with cancer diagnosis as some healthy cells also make these chemicals. Moreover, some non-cancer conditions can also cause high levels of tumor markers.

Some other blood tests might find proteins or other substances made by the cancer and are performed after cancer diagnosis such as:

1. Cancer antigen tests: To assess if the treatment is working. Examples of cancer antigens include prostate-specific antigen (PSA) for prostate cancer and cancer antigen CA-125 for ovarian cancer, carcinoembryonic antigen (CEA) for colon cancer, and alpha-fetoprotein for testicular cancer.
2. Circulating tumor cell tests: To look for cancer cells that might be in the blood when cancer cells are broken away from where they started and are spreading to other parts of the body. Often used in breast cancer, colon cancer, and prostate cancer.
3. Genetic tests: These tests look for small pieces of cancer cells’ DNA that make their way into the blood. Genetic tests are used in cancer patients to understand the DNA changes present in the cancer cells and the results can be considered to select the best treatment.

Cancer diagnosis has been swiftly improving due to advancements in technology and the progresses in our understanding of the disease. As a result, a reliable diagnostic approach could be feasible in near future. The premise of future advancements in cancer research is to use blood samples to test DNA changes to detect signs of cancer in healthy people with no symptoms. A cancer blood test can screen the blood of cancer patient for the traces of released DNA by the dying tumor cells. Therefore, a highly desired and ideal diagnostic approach is a blood test that could detect cancer at its early onset with high accuracy. A cancer blood test to detect the early signals of cancer would be an alternative to invasive procedures like tissue biopsies and provides the patients with major benefits including receiving treatment earlier with a higher chance of success in case of positive test or ruling out the cancer with no need for invasive procedures. A cancer blood test with high accuracy will also allow targeted diagnostic evaluations at the onset of cancer. The research conducted here is in-line with an active area of research to use a blood sample for testing DNA changes for cancer diagnosis. Our research using paired tumor-blood samples taken from lung cancer patients is an essential step towards this goal. We demonstrated potential differences and similarities in DNA copy numbers in tumor sample in comparison with blood sample that can be potentially used in future to develop cancer tests for lung cancer diagnosis using patient’s blood sample.
